# Evaluation of an adult inpatient eating disorders unit

**DOI:** 10.1186/2050-2974-1-S1-P3

**Published:** 2013-11-14

**Authors:** Sue Patterson, Warren Ward, Jeremy Johnson, Rachael Bellair, Shane Jeffrey

**Affiliations:** 1 Royal Brisbane and Women's Hospital, Australia; 2 University of Queensland, Australia

## Introduction

Whilst most treatment for eating disorders occurs in an outpatient setting, inpatient treatment and care can play an important role if a patient is medically unstable, or is not responding to outpatient or day program treatments. With limited research conducted to date, the effectiveness of inpatient care and the role it plays in promoting recovery remain incompletely elucidated.

## Study Context

The Royal Brisbane and Women's Hospital (RBWH) Eating Disorders Unit (EDU), one of few public adult inpatient units for eating disorders in Australia, comprises five beds in a general psychiatric ward. Treatment is provided by a multi-disciplinary team. The goals of inpatient treatment include medical stabilisation, nutritional rehabilitation with restoration of weight (Body Mass Index (BMI)>17), promoting psychological recovery and linking patients with outpatient treatment.

## Method

We conducted a retrospective descriptive study using data collected routinely at the RBWH EDU between October 2008 and October 2012. Psychopathology was evaluated using the Eating Disorder Examination (EDE), Eating Disorder Inventory (EDI), and Depression Anxiety Stress Scale (DASS). Patients and carers were also asked to provide feedback on satisfaction of care.

## Results

During the 4-year period of study there were 109 admissions (104 female, 5 male) of 72 patients. Admission diagnoses were Anorexia Nervosa (AN, 58%), Bulimia Nervosa (3%), and Eating Disorder Not Otherwise Specified (40%). Mean duration of stay was 46 days (range 2 to 186). Body Mass Indeces (BMIs) at admission were between 11.6 and 27.3 (mean of 14.9 for AN) and increased significantly (*p*<.01) at discharge by 1.6 (2.1 for AN). Psychopathology was significantly decreased at discharge on all EDE and DASS scales and on 14 of 18 EDI scales. A high level of satisfaction with the RBWH EDU program was reported by both patients and carers.

